# VennMaster: Area-proportional Euler diagrams for functional GO analysis of microarrays

**DOI:** 10.1186/1471-2105-9-67

**Published:** 2008-01-29

**Authors:** Hans A Kestler, André Müller, Johann M Kraus, Malte Buchholz, Thomas M Gress, Hongfang Liu, David W Kane, Barry R Zeeberg, John N Weinstein

**Affiliations:** 1 Neural Information Processing, University of Ulm, Germany; 2 Internal Medicine I – Gastroenterology, University Hospital Ulm, Germany; 3 Department of Gastroenterology and Endocrinology, University Hospital of Marburg, Germany; 4 Georgetown University, Washington, DC, USA; 5 SRA International, USA; 6 National Institutes of Health, National Cancer Institute, Laboratory of Molecular Pharmacology, Genomics and Bioinformatics Group, USA

## Abstract

**Background:**

Microarray experiments generate vast amounts of data. The functional context of differentially expressed genes can be assessed by querying the Gene Ontology (GO) database via GoMiner. Directed acyclic graph representations, which are used to depict GO categories enriched with differentially expressed genes, are difficult to interpret and, depending on the particular analysis, may not be well suited for formulating new hypotheses. Additional graphical methods are therefore needed to augment the GO graphical representation.

**Results:**

We present an alternative visualization approach, area-proportional Euler diagrams, showing set relationships with semi-quantitative size information in a single diagram to support biological hypothesis formulation. The cardinalities of sets and intersection sets are represented by area-proportional Euler diagrams and their corresponding graphical (circular or polygonal) intersection areas. Optimally proportional representations are obtained using swarm and evolutionary optimization algorithms.

**Conclusion:**

VennMaster's area-proportional Euler diagrams effectively structure and visualize the results of a GO analysis by indicating to what extent flagged genes are shared by different categories. In addition to reducing the complexity of the output, the visualizations facilitate generation of novel hypotheses from the analysis of seemingly unrelated categories that share differentially expressed genes.

## Background

A major goal, as well as a major challenge, of transcriptome analyses is the interpretation of results in a biological context. In many comparative studies, the primary results of the analyses are lists of genes expressed differentially between different groups of samples. The identification of underlying biological themes (e.g. alterations of specific pathways, triggering of complex cellular responses, activation of specific transcriptional programs) is usually not straightforward. By providing a controlled and structured vocabulary for the functional description of gene products, the Gene Ontology (GO) database [1] represents a useful resource for comprehensive functional annotation of gene lists. Moreover, GO categories that are significantly enriched in the differentially expressed genes can be identified, providing clues to the biological causes and consequences of observed transcriptome changes. Since genes and gene products are usually associated with several GO terms, such an analysis tends to increase, rather than reduce, the information load. Methods are therefore needed to structure and adequately visualize the results of a GO analysis (e.g., by indicating to what extent genes are shared by different categories). In addition to simply reducing the complexity of the output, such visualizations may facilitate the generation of novel hypotheses from observation of seemingly unrelated categories that share differentially expressed genes.

Diagrammatic notations involving circles and other closed curves have been used to represent classical syllogisms since the Middle Ages [2]. In the 18th century the mathematician Leonhard Euler introduced the notation that is now called the "Euler diagram" to illustrate relationships among sets. That notation uses the topological properties of enclosure, exclusion, and partial overlap to represent the set-theoretic concepts of containment, disjointness, and intersection. Another notation was invented by John Venn in the 19th century. A Venn diagram contains *n *closed curves representing *n *sets, in which all sets must intersect. Those diagrams rarely provide a useful visual representation if five or more sets are involved (in general using non-oval contours). Moreover, it can be shown that Venn diagrams with circles are not generally possible for more than three sets. Here, we relax the requirement of total intersection of all curves, limit ourselves to circles, but impose the additional requirement that area must be as nearly as possible proportional to set size. The last restriction enables us to visualize the set relationships at least semi-quantitatively. The problem of proportional areas is in general not perfectly solvable (i.e. fulfilling all requirements of containment, disjointness, and intersection with set size proportional to the corresponding area). Rather, the aim is to construct approximate solutions. A preliminary report [3] described a basic implementation of those ideas. We now describe how the analytical ideas can be used to construct Euler/Venn diagrams, together with full, seamless integration into GoMiner.

### Finding interesting intersections

The Gene Ontology (GO) database imposes three hierarchically structured ontologies, or classification systems, on gene products:

• Molecular function – an activity at the molecular level (e.g. catalytic/transporter activity or binding).

• Biological process – a series of molecular functions (e.g. signal transduction).

• Cellular component – an anatomical structure (e.g. rough endoplasmic reticulum or nucleus) or a gene product macromolecular structure (e.g. ribosome, proteasome or a protein dimer).

Because each GO category may have more than one parent, the hierarchy takes the form of a directed acyclic graph (DAG), with edges pointing from a parent (a more general category) to children (more specific categories). The three major ontologies share no nodes and are therefore independent DAGs. Each gene product is associated with one or more categories. The root subsumes all three ontologies and is therefore associated with all categorized gene products in the database.

GoMiner [4-6] evaluates the significance of each GO category by a Fisher's exact p-value and a false discovery rate (FDR) to detect differentially expressed genes of a microarray assay that are significantly over-represented in a certain GO category.

One analytical approach is to select from the GO DAG an interesting subset of categories that meet two filtering criteria:

• the *p*-value or the FDR does not exceed a threshold, and

• the number of genes in a category lies in a pre-specified range of interest, since categories that are too small (containing only a few genes) or too large (such as the whole ontology) may be considered uninformative.

**Figure 1:**
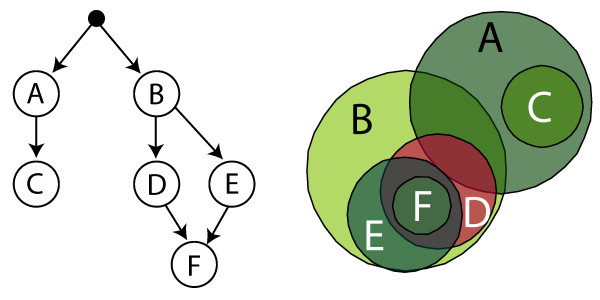
**DAG vs Euler diagram representation: Non-trivial set relations**. Comparison of a directed acylic graph (DAG) representation (left diagram) and an area-proportional Euler diagram visualization (right diagram) of the same set family. From the (hypothetical) Gene Ontology DAG on the left side the following set relations can be inferred: *C *⊆ *A*, *D *⊆ *B*, *E *⊆ *B*, *F *⊆ *D*, *F *⊆ *E*, and *D *∩ *E *≠ ∅ since the two nodes have at least the elements of *F *in common. The Euler diagram representation reveals, in addition to the approximate visualization of the set cardinalities, further set relations (*A *∩ *B *≠ ∅, *C *∩ *B *= ∅, *D *∩ *A *≠ ∅, *E *∩ *A *= ∅, and *F *∩ *A *= ∅) that are not specified in the DAG representation.

The subset of nodes selected from the DAG according to those criteria may be unconnected, the result being a forest of DAGs. The observation of two or more significant categories for which a direct path exists – so that they are in a child/parent relationship of a certain degree may be less surprising than the same observation for a more distant (e.g., cousin) relationship such as the intersection of A and D in Figure 1. In a subsequent step, the set structure of the differentially expressed genes can be analyzed for the previously selected nodes. That is an impractical task for more than two gene sets. The area-proportional Euler diagram approach was developed for that type of analysis. Every category is represented by a circle (polygon) with an area approximately proportional to the number of elements in the set. Intersection, non-intersection, and containment relationships among sets are easily readable with Euler diagrams, but the number of categories in the Euler diagram has to be limited since showing more than about 10 categories in a single diagram is computationally and visually infeasible.

### Reasoning with area-proportional Euler diagrams

**Figure 2:**
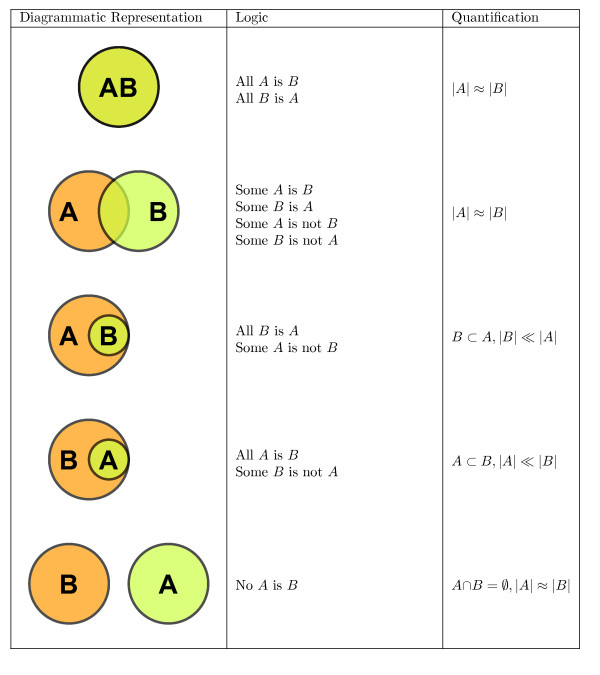
**Reasoning with area-proportional Euler diagrams**. List of possible inferences for two sets.

Basic set relations such as inclusion, exclusion, and containment can easily be visualized with Euler diagrams in a topologically rigorous way allowing for the inference of secondary information from otherwise complex set relations (see Figure 2, compare [[7], Ch. 2]). This situation is demonstrated in the following example:

The scenario of Figure 1 (right) could be described by the following syllogisms:

**(a1) **All *C*s are *A*s

**(a2) **No *C*s are *B*s

**(a3) **Some *A*s are *B*s

**(a4) **All *D*s and *E*s are *B*s

**(a5) **All *F*s are *E*s and *D*s

**(a6) **Some *E*s are *D*s

**(a7) **No *E*s are *A*s

**(a8) **Some *D*s are *A*s

¿From the Euler diagram representation or the previously defined rule set the following relations can be inferred:

**(a5) + (a4) + (a7) **⇒ **(b1) **No *F*s are *A*s

**(a2) + (a4) + (a5) **⇒ **(b2) **No *F*s are *C*s

**(a2) + (a4) **⇒ **(b2) **No *D*s are *C*s

In addition, the (approximate) area-proportionality enables assessment of the number of elements in the sets, and leads to the following inferences:

**(c1) ***|A| *≈ *|B|*

**(c2) ***|E| *≈ *|D|*

**(c3) ***|C| *<*|E| *and *|C| *<*|D|*

**(c4) ***|F| *<*|C|*

**(c5') ***|D *∩ *B*| < |*A *∩ *B|*

In the last conclusion (c5') has to be verified by observing the exact cardinalities as the overlaps need not to be strictly proportional to the area, as the visualization depends on the concrete set family and the parameters of the cost functional. It is important to control the existence of missing intersections if the Euler arrangement is not able to express fully all set relations. However, missing intersections occurred for GO data.

## Results

### Visualization results

**Figure 3:**
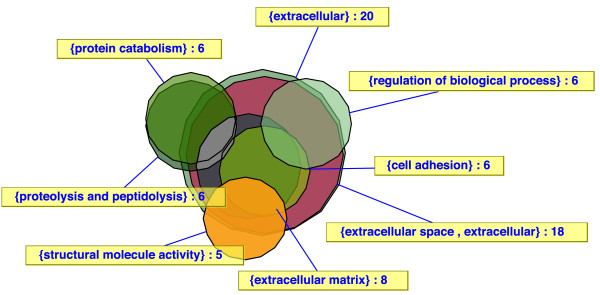
**Euler diagram of results from a microarray experiment comparing stellate cells with fibroblasts**. A visualization of gene sets differentially expressed between specialized mesenchymal cells (stellate cells) and normal skin fibroblasts (minimum total number of genes per category: 100; max p-value: 0.025) is shown [8]. Although a total of 8 ontology categories were reported to be significantly enriched among the changed genes, the analysis revealed that those categories strongly overlap and form a single large cluster of cell surface/extracellular matrix-related categories. The corresponding directed acyclic graph is not shown. None of the categories (except for extracellular space/matrix as a descendant of extracellular) form direct or indirect parent/child relationships.

The study in Figure 3 used 23000 feature 'whole genome' arrays to identify genes differentially expressed between stellate cells (specialized mesenchymal cells) and normal skin fibroblasts. The list of differentially expressed genes was compared with the list of all genes exceeding a minimum expression threshold (normalized expression value greater 0.5 in at least one of the sample sets) to identify GO categories significantly enriched with differentially expressed genes [8]. Since the tree representation was complex, we visualized the results of the analysis with an Euler diagram. It shows the overlap of genes in different GO categories resulting from the association of genes with multiple GO categories (see Figure 3). The principal GO categories identified as significantly enriched with differentially expressed genes in the GoMiner analysis included very diverse and seemingly unrelated GO terms such as 'structural molecule activity', 'cell adhesion' and 'protein catabolism'. The Euler/Venn visualization approach, however, revealed that those categories strongly overlapped and fell within a single cluster of categories connected by their mutual content of cell surface and extracellular matrix genes. That observation led to the conclusion that the largest difference between the cell types under investigation is their distinct and highly specialized contribution to the production of connective tissue [8].

### Simulation results

**Figure 4:**
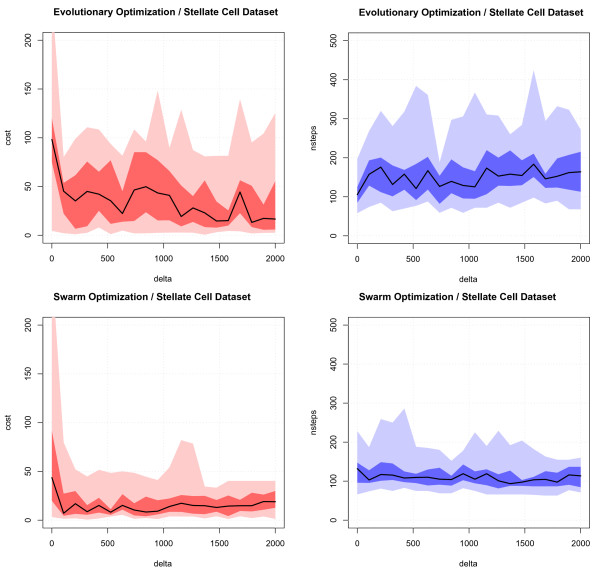
**Particle swarm optimization vs evolutionary strategy**. To compare the two optimization strategies and to show that the non-compactness penalty (*d*, see Section Cost function) leads in many cases to a better convergence (lower original error term E), we computed Euler diagrams for one previously published gene expression dataset containing genes differentially expressed between a specialized mesenchymal cell type (stellate cells) and normal skin fibroblasts (filter settings: minimum total: 40; maximum total: 140; max p-value: 0.05). For different delta values (= weight of the pressure term) the cost functional E is shown for the two optimization strategies. The number of optimization steps until the stop criterion (maximum number of constant steps = 50, maximum number of steps = 500) is met is shown on the right side. Min/max (light color), interquartile range (dark color), and median value (black line) over n = 20 runs and 20 different delta parameter settings (x-axis) ranging from 0 to 2000 are shown.

Generally, the requirement of area being proportional to set size cannot be fulfilled for all configurations. The deviation from optimality is measured by an error function (see Methods section). The proposed error function evaluates the goodness of the graphical Euler arrangement by putting different weights on the (contradictory) constraints. Since an arrangement may become unconnected, a compactness term was used (as part of the cost function) to weight compact solutions more strongly. To show that in many cases the compactness term leads to a better convergence (lower original error term E), we computed Euler diagrams for 10 artificial random data sets (see supplementary information [9]) and the data set from Buchholz & Kestler et al. [8] (see Figure 4; GO filter settings: minimum total: 40; maximum total: 140; max p-value: 0.05 – the complete data are available at [9].

We also compared the two biologically inspired optimization strategies, evolutionary optimization (EO) and particle swarm optimization (PSO). The quality of the solution was assessed by the cost function term ("E" see Methods section) and the number of optimization steps required to reach a stable solution. Toward that end, we varied the parameters of two different optimization algorithms. We used 20 different settings for the compactness term (delta parameter; see Methods), equally spaced in the range [0, 2000], with 20 runs (using different seed values for the random number generator) for each data set. Further, the number of individuals (EO) or particles (for the PSO) was set to 50 with a maximum of 500 iterations. If the best individuals/particles could not improve the cost function within 50 iterations the optimization was stopped.

For the stellate cell data set (import parameters: minimum category size 40, maximum category size 140, maximum p-Value of 0.05) the evaluation resulted in a total of 400 simulations for each of the two optimization strategies (EO versus PSO). An unpaired one-sided Wilcoxon rank sum test revealed significantly lower cost function values (*p *< 2.2 · 10^-16^) and a significantly lower number of iteration steps (*p *< 2.2 · 10^-16^).

In addition to testing of the real world data set, we performed simulations on 10 random set families (a total of 4000 simulations for each algorithm, details of performing the simulations are given in the supplementary information [9]). Each of those 10 families consisted of 5 sets. The pooled results (for both algorithms) gave a *p*-value of 4.567 · 10^-10 ^for the value of the cost function, and a *p*-value below 2.2 · 10^-16 ^for the number of iteration steps (both unpaired one-sided Wilcoxon rank test).

## Discussion and Conclusion

Analyzing functional annotations of genes and gene products is becoming increasingly important in the comprehensive GO analysis of microarray data. Identification of functional interrelations between differentially expressed genes detected by GoMiner contributes substantially to uncovering fundamental biological programs and superordinate pathways reflected in the transcriptional differences. Displaying the results of such analyses has remained challenging.

We have introduced a new method for visualizing annotated gene sets as overlapping circles in the plane. The approach is loosely related to other procedures such as Venny [10] (4-set Venn diagrams, no area proportionality) and Tree-EASE [11,12], (which uses hierarchical clustering), or GoMiner to find functionally related genes, which are then annotated. Our focus is on a semi-quantitative visualization that could be performed after such analyses. Although there is in general no perfect solution for these area-proportional Euler diagrams using circles or regular polygons, the proposed approach leads to easily interpretable visualizations.

We draw diagrams with zero size zones that are shaded, in accord with the original visualization of Venn diagrams [13]. The proposed type of Euler diagram is appropriate only for problems involving a relatively small number of intersections, a situation that often pertains to data originating from the GO database, since those data are naturally hierarchically structured. Area-proportional Euler diagrams are, in most cases, a trade-off between accuracy of the intersection areas and meaningful polygon arrangements without missing faces (= inconsistencies) and without too many empty faces (which are shaded). Therefore, we suggest several alternative formulations of the cost function to focus on different aspects of the data, such as the importance attached to intersections involving many sets (weights *w*_*k*_) and the importance attached to giving equal weight to elements (genes) or groups (GO categories) (see error function *f*_1 _versus *f*_2_, respectively, in the Methods section).

The simulations produced the rather unexpected result that the PSO outperformed the EO, both in generating solutions with a lower cost and in faster convergence. The momentum inherent in the PSO seems to be better suited to the graphical optimization situation. A possible further improvement could be achieved by using a gradient descent optimization (similar to those proposed in [14]) for fine-tuning a coarse solution from the evolutionary strategy. Gradient descent alone is not able to find the optimal solution, since for more than 3 sets, local minima exist in the error function. On the other hand, it is impractical to differentiate the cost function analytically, and an approximation of the gradient is computationally expensive (compare the complexity estimation in the Methods section). Therefore, a gradient descent algorithm seems not to be particularly appropriate for this problem.

In summary, we have developed a method for visualizing set relationships that extends the inferences that can be expressed by DAGs. Intersections in different branches can now be visualized. The approach is implemented as an interactive application specifically designed for use with GoMiner in the context of the GO database. It has been integrated directly into the original GUI GoMiner software and is compatible with High-Throughput GoMiner.

## Methods

It was demonstrated by Chow and Ruskey [14,15] that the task of visualizing intersecting sample sets by area-proportional Euler diagrams is in general not perfectly solvable for more than two sets with circles in the plane. We therefore defined a cost function reflecting the conflicting constraints of circle overlap and cardinality of the intersection set and sought the best compromise solutions employing evolutionary and swarm approaches for optimization [for details see Additional file 1].

### Cost function

We propose a cost functional *E *mapping the regular polygon (or circle) centers to an error value describing the goodness of the solution. The function *E *includes a trade-off between the correct graphical intersection areas and the true set sizes. The problem is first partitioned into disjoint, independently solvable subproblems. That can be accomplished by finding the connected components of an intersection graph that has one vertex for each set and edges that connect intersecting sets. The connected components can be found using a depth-first search (compare [16,17]) which takes *O*(*n *+ *m*) steps, where *n *is the number of vertices (sets) and *m *is the number of edges (which can be at most *n*(*n *- 1)/2). The resulting complexity is *O*(*n*^2^) to partition the problem. In the following it is therefore assumed that all sets have at least one intersecting partner. Let *A*_1 _...*A*_*m *_⊆ U be a sequence of intersecting subsets of the overall gene set U and let *G*_1 _...*G*_*m *_⊆ ℝ^2 ^be a graphical two-dimensional representation of the sets.

For a perfectly solvable case the cardinality of every intersecting subset *A*(*I*) = ∩_*i∈I *_*A*_*i *_for *I *⊆ {1 ... *m*} is proportional to its corresponding graphical intersection area *G*(*I*) = *η*area (∩_*i∈I *_*G*_*i*_) so that |*A*(*I*)| = *G*(*I*) for a proportionality factor *η *> 0. Define *A*(∅) = U. A possible error function, which was implemented in Kestler et al. [3], is *E *= ∑_*I *⊆ {1...*m*}_*w*_|*I*|_*f *(*I*) with the partial errors

$$f_{1}(I)=d(G(I),\left|A(I)\right|)\{\begin{array}{lll} \alpha & \text{if }A(I)=\emptyset & \\ \beta & \text{if }A(I)\neq \emptyset , & G(I)=0\\ \gamma & \text{otherwise} & \end{array}$$

with a distance function *d*(*g*, *c*) and constants *α*, *β*, *γ *≥ 0 allowing different weights on the three cases: unwanted graphical overlaps, missing graphical intersections, and area deviations.

**Figure 5:**
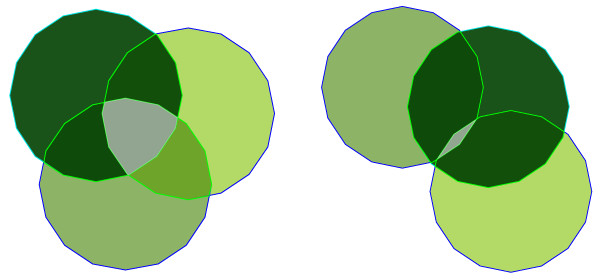
**Influence of different parameter settings in the cost functional**. Example with the three sets *A *= {1, 2, 3}, *B *= {2, 4, 5}, and *C *= {3, 4, 6} such that there is a pairwise overlap of one element, but not among all three sets, i.e. |*A *∩ *B| *= |*A *∩ *C| *= |*B *∩ *C| *= 1 and |*A *∩ *B *∩ *C| *= 0. That means no perfect solution satisfying all constraints can be found, but only approximate ones, which are influenced by the relative weighting of the partial costs. The left diagram shows the settings *α *= 0 and *β *= *γ *= 1 so that unwanted intersections (the gray area in the middle) are ignored. For the diagram on the right, *a *was set to 50, thus showing a smaller gray area at the expense of a larger area deviation.

The effect of using different parameter settings to highlight different aspects of the data is demonstrated in Figure 5. The weights *w*_*k*_, *k *= 1 ... *m *were previously chosen to be *w*_*k *_= 1/(*k *- 1) (for *k *> 1) and *d*(*g*, *c*) = (*g *- *c*)^2^.

Error function *f*_1 _weights the (potentially large) intersection of two large sets much stronger than the (potentially small) intersection of two small sets or a small with a large set. Since the intersection size of a sequence of sets is restricted by its smallest set, we propose to normalize the partial errors by this upper bound. Furthermore *f*_1 _does not take into account that a barely visible intersection (where an intersection should be present) is worse than a little too much graphical overlap (compare Figure 5). An error function compensating for those two effects can be defined by

$$f_{2}(I)=\frac{d(G(I),\left|A(I)\right|)}{\min_{i\in I}\left|A_{i}\right|}\{\begin{array}{lll} \alpha & \text{if }A(I)=\emptyset & \\ \beta & \text{if }A(I)\neq \emptyset , & G(I)<\left|A(I)\right|\\ \gamma & \text{otherwise} & \end{array}.$$

In general one should select *β *> *γ*.

Now we put a further constraint on the error weights *w*_*k *_in *f*_1 _such that the intersection of *k *sets must be at least as important as the intersection of > *k *sets. That constraint can be formulated by the condition

$$\sum_{I\subseteq \{1...m\},\left|I\right|=k}\hat{E}(I)\geq \sum_{S\subseteq \{1...m\},\left|S\right|>k}\hat{E}(S)$$

where $\hat{E}$(*I*) is defined to be an upper bound for the cost for the intersection *I *⊆ {1 ... *m*}. That condition dictates that the maximum possible error of intersecting > *k *sets must be smaller than the maximum possible error of intersecting *k *sets. Let *M *be an upper bound of the partial error *f*_1 _in the above case. *M *could be max_*i *_*d*(0,|*A*_*i*_|)/|*A*_*i*_| max{*α*, *β*, *γ *}. So equation above can be formulated as

$$\begin{array}{cc} \left(\begin{array}{c} m\\ k \end{array}\right)w_{k}M\geq \sum_{j=k+1}^{m}\left(\begin{array}{c} m\\ j \end{array}\right)w_{j}M, & i\leq k<m \end{array}$$

The conditions can be fulfilled (such that equality holds) by finding weights from the backside starting with *w*_*m *_= 1 down to *w*_1_:

$$\begin{array}{cc} w_{k}=\frac{2^{m-k-1}}{\left(\begin{array}{c} m\\ k \end{array}\right)}, & 1\leq k<m \end{array}.$$

An error function evaluation requires *O*(*Lm*2^*m*-1^) computation steps when using polygons with *L *edges (intersecting two polygons with *M *and *N *edges can be computed in *O*(*M *+ *N*) with O'Rourke's algorithm [18]). For problems with ≥ 8 categories the complexity may be reduced due to time and space limitations by observing only intersection sets *I *with *|I| = K *for an upper bound *K*. The probability that for a highly intersecting group of sets a perfect diagram exists (up to a high level of intersections) is nevertheless very low. The size of an Euler diagram is defined as the number of faces this diagram should have to reflect all intersections occuring in the data

*e*(*I*) = |{*I *⊆ {1 ... *m*}|*A*(*I*) ≠ ∅ }|

The current implementation enables one to observe the partial error *f *for each intersection set *A*(*I*), *I *⊆ {1 ... *m*}. In the following we propose some extensions of the previous visualization scheme:

**i) **To allow for better adaption and reduction of the unwanted regions (marked in gray), the solution space was extended by allowing the optimization to vary the polygon areas in a certain range such that the order conditions *area*(*G*_*π*(1)_) ≤ ... ≤ *area*(*G*_*π*(*m*)_) were preserved with a permutation *π *such that |*A*_*π*(1)_| ≤ ... ≤ |*A*_*π*(*m*)_|. Sets with equal cardinalities were represented by equal graphical areas. Only radial scaling of the polygons was allowed. Unfortunately, this strategy did not improve the visual representation even though the current implementation neglected the order criteria and so had more freedom to adapt (the solutions found were certainly not more informative).

**ii) **If using many sets, the scaling must be chosen small in order to fit the polygons into the unit box [0, 1]^2^. Therefore, the empty space in relation to the polygon areas will be very large, and the optimization may take a long time or may not produce a plausible solution (in those cases, the graphical representation is unconnected).

Therefore the cost function was extended with a further penality term

$$\hat{E}=\sum_{i<j}\left||a_{j}-a_{i}||\cdot ||A_{j}\cap A_{i}|-\text{area}(G_{j}\cap G_{i})\right|$$

with the polygon centers **a**_1 _...**a**_*m *_∈ ℝ^2^, weighting compact solutions higher than scattered arrangements. If the graphical intersection is perfect, the second term in the sum becomes zero, and the penality term behaves neutrally. Otherwise, the term drives the polygons (which should intersect) towards each other until they eventually meet. The total error functional then computes to

$$E^{\prime }=E+\delta \hat{E}$$

with the weighting parameter *δ *≥ 0.

To avoid local minima, the cost function *E *is minimized over the polygon centers (shape and orientation of the polygons remain fixed) using a swarm optimization algorithm [19] and an evolutionary strategy with self-adapting mutation rates [20].

### Particle swarm optimization

Particle swarm optimization (PSO) [19] is a biologically motivated optimization technique similar to evolutionary strategies [20] and genetic algorithms. A swarm consists of a number *N *of interacting particles such that each particle *j *= 1 ... *N *represents a solution **x**^(*j*) ^∈ ℝ^*n *^in the *n*-dimensional space having fitness *f*(**x**^(*j*)^). Additionally, each particle has a velocity vector **v**^(*j*) ^∈ ℝ^*n *^that specifies its current movement in space for each axis. In the beginning, at *t *= 0, the locations are chosen uniformly from the *n*-dimensional unit hypercube [0, 1]^*n *^to which this constrained optimization problem is scaled. The velocities are chosen independently from the range [*-v*_*max*_*, v*_*max*_] for a constant *v*_*max*_> 0. The swarm evolves over time in discrete time steps with update rules for the locations

$$x_{t+1}^{\left(j\right)}=x_{t}^{\left(j\right)}+v_{t}^{\left(j\right)}$$

and velocities

$$v_{t+1}^{\left(j\right)}=v_{t}^{\left(j\right)}+c_{glob}U[0,1](x_{t}^{\left(glob\right)}-x_{t}^{\left(j\right)})+c_{loc}U[0,1](x_{t}^{\left(j,loc\right)}-x_{t}^{\left(j\right)})$$

with the positive acceleration constants *c*_*glob *_and *c*_*loc*_. The global best solution (having the maximum fitness value) among all particles for all *t' *≤ *t *is defined as $x_{t}^{\left(glob\right)}$; the local best solution for a single particle *j *is $x_{t}^{\left(j,loc\right)}$. The velocities are restricted to the interval [*-v*_*max*_*, v*_*max*_] for each axis. The interaction among particles is regulated by the influence of the global and local optima on the velocity term. U [0, 1) is a uniform random variate generating a number from the interval [0, 1). One variation of the method is to restrict the locations to the bounding box [0,1]^*n *^and to change the sign of velocity components of the respective dimensions such that the particles bounces back from the wall.

### Evolutionary optimization

A generation contains *N *individuals each representing a permissible solution of the problem. In the following step each individual is mutated, and its fitness is evaluated by the previously defined cost function *E*. An individual is replicated with frequencies proportional to its fitness rank, thus generating offspring until the original generation size is reached. The best individual is always transferred unchanged into the new generation. The process of mutation and replication is repeated until the best individual does not change over a certain number of steps.

An individual consists of a parameter vector $v_{1}^{t}\cdots v_{m}^{t}\in {\mathbb{R}}^{2}$ representing the polygon centers and a vector *σ*^*t *^∈ ${\mathbb{R}}_{+}^{m}$ describing the mutation rate for each parameter. The first population is initialized with uniformly distributed random values such that each parameter stays in a certain range i.e. the polygons must be enclosed by the bounding box [0, 1]^2 ^and the mutation parameters have to be contained in the interval [*τ*_*lower*_, *τ*_*upper*_] with 0 <*τ*_*lower*_<*τ*_*upper*_.

In the mutation step the mutation parameters themselves are mutated

$$\begin{array}{cc} \sigma_{i}^{\left(t+1\right)}=\sigma_{i}^{t}e^{N(0,\tau )} & i=1...m \end{array}$$

and restricted to the interval [*τ*_*lower*_, *τ*_*upper*_]. The constant meta-mutation parameter *τ *> 0 is a pre-specified constant. Then the locations of the polygons are updated

$$\begin{array}{cc} v_{i}^{\left(t+1\right)}=v_{i}^{t}+\left[\begin{array}{c} N(0,\sigma_{i}^{\left(t+1\right)})\\ N(0,\sigma_{i}^{\left(t+1\right)}) \end{array}\right] & i=1...m \end{array}$$

where N(0, *s*) represents a normally distributed variate with mean 0 and variance *σ*. After each mutation, all parameters are restricted to meet the above conditions.

Evolutionary selection and offspring generation are performed by assigning each individual a rank *r *= 1 ... *N *according to its fitness as determined according to the value of the cost functional *E *or *E' *such that the best individual (the one with the lowest cost or highest fitness) has *r *= 1. Each individual is then replicated a number of times inversely proportional to its rank value. Therefore, an individual with rank *r *will have at most [*qN*/*r*] (for a fixed 0 <*q *< 1) offspring. Starting with the highest rank *r *= 1 the new population is filled up until the size *N *is reached and the new generation is complete. All but the first individual (the fittest of the last generation) are mutated.

The optimization process is stopped when the cost functional of the best individual does not improve over a certain number of steps or the number of generations exceeds an upper bound.

### Implementation of VennMaster

The visualization approach was implemented as a platform-independent open source Java application, which is available online [9]. The application allows interactive exploration of Euler diagrams was tested under Windows XP, Linux, and Mac OS X using the Java Runtime Environment 1.5 [21].

When one touches a polygon with the cursor, its area is highlighted and the involved group names and the cardinality of the intersection set are shown. Among many other parameters involving the evolutionary strategy and the error function, the number of edges of the polygons can be configured. Those settings can be exported and imported in XML format [22]. Furthermore, a gene list of the selected intersection set(s) is shown in an information field. Unresolved intersections (for which no corresponding polygon intersection exists) are listed in the field "Inconsistencies". For each set or intersection set, a text label can be attached (see Figure 3). Labels and polygons can be moved by drag and drop (the cost function will be updated immediately). So the user can interactively modify the configuration and may restart the optimization process on the changed arrangement. Set positions can be locked so that they will not be moved by the optimizer. The optimization process can be controlled via a parameter dialog (see supplementary information [9]). The area-proportional Euler diagrams may be saved as JPEG or SVG (Scalable Vector Graphics (SVG) is a XML based graphics format [23]).

### Integration with GoMiner

GoMiner [4] is a tool for biological interpretation of "omic" data – including data from gene expression microarrays. Omic experiments often generate lists of dozens or hundreds of genes that differ in expression between samples. GoMiner uses the Gene Ontology to identify the biological processes, functions and cellular components represented in these lists, and groups the genes into biologically-coherent categories. The ability to import files from GoMiner was included in the software to permit analysis of functional categories of differentially expressed genes. For the GoMiner files, categories were pre-filtered so that the number of genes in the included categories would lie in a certain definable range and would not exceed a given FDR or *p*-value. Alternatively, a simple tab delimited file format with an element/group pair in each line can be used as input. The current version enables the export of an error profile listing all partial errors for each non-empty set combination *I *⊆ {1 ... *n*} such that *G*(*I*) > 0 or *A*(*I*) ≠ ∅. Each line contains the values *I*, *|I|*, *G*(*I*), *|A*(*I*)*|*, and *f*(*I*).

**Figure 6:**
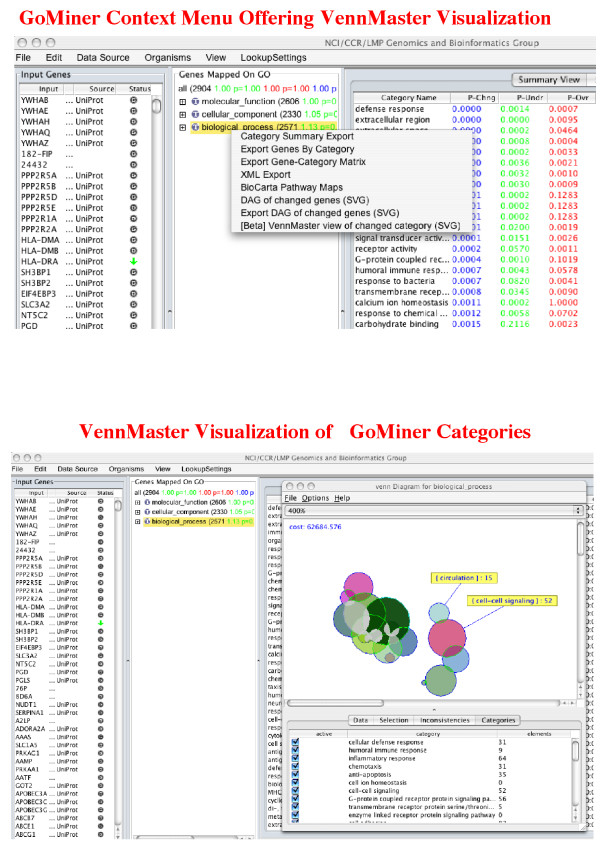
**Implementation: GoMiner integration**. Context menu in the integrated VennMaster-GoMiner user interface. In this example, VennMaster is about to be applied to visualization of the GoMiner categories in the "biological process" branch of GO. The result of completing the step is shown in the lower panel.

VennMaster was recently integrated into the GoMiner software (see Figure 6). That integration provides a seamless interface for the user, and eliminates the overhead of performing file I/O operations and managing external files. As part of a pilot project, GoMiner is being integrated into the Cancer Biomedical Informatics Grid initiative [24,25], which aims to provide a grid connecting individuals and institutions to facilitate the interoperable sharing of data and informatic tools. Integration of VennMaster and GoMiner will result in the integration of VennMaster in caBIG, thus enhancing both the availability of VennMaster to the broad user community, and the ease with which it can be used.

## Availability and requirements

The visualization scheme is available as a platform-independent Java application (> JRE 1.5.0) from [9]. VennMaster is also now directly integrated into the GoMiner application (see Figure 6) available at [6] for direct Euler diagram representation of GO categories enriched with flagged genes.

## Authors' contributions

HAK designed the study and drafted the manuscript. AM participated in the design of the study, implemented the software and helped to draft the manuscript. JMK revised the software and helped to draft the revision. MB participated in the design, evaluated the procedures and helped to draft the manuscript. TMG participated in its design and coordination and helped to draft the manuscript. HL integrated VennMaster and GoMiner. DWK provided overall direction for development of GoMiner. BRZ participated in the design of the study and performed the statistical analysis. JNW participated in its design and coordination and helped to draft the manuscript. All authors read and approved the final manuscript.

## Supplementary Material

Additional file 1 **Implementation details**. Details on the software implementation. Click here for file
