# Supplementary material for: VennMaster: Area-proportional Euler diagrams for functional GO analysis of microarrays
Source: BMC Bioinformatics. 2008 Jan 29;9:67. doi: 10.1186/1471-2105-9-67 (PMC2335321; doi:10.1186/1471-2105-9-67)
Supplement: Additional file 1 — Implementation details. Details on the software implementation. [file 1471-2105-9-67-S1.pdf]

Implementation details for "VennMaster: Area-proportional Euler diagrams for functional GO analysis of microarrays"

Hans A. Kestler*[1,2], André Müller[2], Johann M. Kraus[1,2], Malte Buchholz[2,3], Thomas M. Gress[2,3], Hongfang Liu[4,6], David W. Kane[5], Barry R. Zeeberg[6] and John N. Weinstein[6]

[1]Neural Information Processing, University of Ulm, Germany
[2]Internal Medicine I – Gastroenterology, University Hospital Ulm, Germany
[3]Department of Gastroenterology and Endocrinology, University Hospital of Marburg, Germany
[4]Georgetown University, Washington, DC, USA
[5]SRA International, USA
[6]National Institutes of Health, National Cancer Institute, Laboratory of Molecular Pharmacology, Genomics and Bioinformatics Group, USA

Email: Hans A. Kestler*- hans.kestler@uni-ulm.de. All authors contributed equally;

*Corresponding author

Intersecting convex polygons

The intersection of two convex polygons can be computed in $O(n+m)$ steps using O'Rourke's algorithm [3]. It is assumed that the polygon borders ∂P and ∂Q with m and n points are oriented counterclockwise. After choosing two directed edges A from P and B from Q the subsequent steps involve counterclockwise moves of one of the two edges in order to find all crossings. The algorithm always advances the edge behind to chase the other (waiting) edge (see Figure S1). Define $H(A)$ to be the hyperplane to the left of vector A, and $A \times B$ the crossproduct, which is greater than zero if the shortest turn of A into B is counterclockwise (see Table S1). a and b are the heads of the vectors A and B. The cases $P \cap Q = \emptyset$, $P \subset Q$, and $Q \subset P$ must be handled separately when the algorithm does not succeed in finding a polygon. For those cases it is necessary only to check whether a single point of P lies in Q and vice versa.

Figure S1 - Illustration of the polygon intersection algorithm

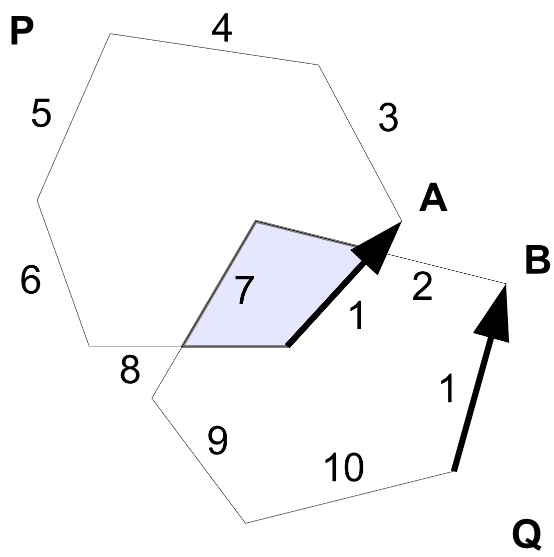

Figure S1: The edges are numbered according to their movements. The starting edges (numbered with 1) are printed in bold.

Table S1 - Rules for the polygon intersection algorithm

$A \times B$	halfplane condition	advance rule
> 0	$b \in H(A)$	A
> 0	$b \notin H(A)$	B
< 0	$a \in H(B)$	B
< 0	$a \notin H(B)$	A

Area calculation of polygons

The polygon areas are computed by applying the Gaussian integration theorem in the plane (see e.g. [4])

$$\int_B \underbrace{\left(\frac{\partial P}{\partial x} - \frac{\partial Q}{\partial y}\right)}_{:=F} d(x,y) = \oint_{\partial B} P dy + Q dx$$

It states that the value of an area integral of the above form (the left side integrates over a scalar field) on a closed domain $B \subset \mathbb{R}^2$ can be expressed by a curve integral along the boundary ∂B (right side).

Both functions $P(x, y)$ and $Q(x, y)$ have to be continous and differentiable. The area $A = \int_B dxdy$ can be calculated with $P(x, y) := x$ and $Q(x, y) := 0$. The right side then evaluates to

$$|B| = \oint_{\partial B} dy$$

The value of this integral will now be expressed as a parametrized curve integral using a closed curve $\gamma(t) \in \mathbb{R}^2$.

$$|B| = \int F(\gamma(t))d\gamma(t)$$

The whole integral is broken up into pieces consisting of the polygon segments in counterclockwise order. Let $(x, y)_{k=1}^{L} \in \mathbb{R}^2$ be a polygon. Each segment of the polygon is parametrized as follows

$$\gamma^{(k)}(t) := \begin{bmatrix} x_k + t \cdot \Delta x_k \\ y_k + t \cdot \Delta y_k \end{bmatrix}$$

where $\Delta x_k = x_{k+1} - x_k$, $\Delta y_k = y_{k+1} - y_k$, $x_{L+1} = x_1$, $y_{L+1} = y_1$ and $t \in [0, 1]$.

For the segment $\gamma^{(k)}$ the partial integral A_k $(k = 1 \ldots L)$ evaluates to

$$A_k = \int_{\gamma^{(k)}} xdy$$

which can be expressed with the parametrized curve $\gamma^{(k)}$ as

$$\int_0^1 \gamma_x^{(k)}(t)\dot{\gamma}_y^{(k)}(t)dt = \int_0^1 (x_k + t\Delta x_k)\Delta y_k dt$$

which simplifies to

$$A_k = x_k \Delta y_k$$

After some conversions the area then computes to

$$A = \sum_{k=1}^{L} A_k = \sum_{k=1}^{L} x_k(y_{k+1} - y_k) \quad , y_{L+1} := y_1.$$

A is positive for polygons with a counterclockwise orientation and negative for those with a clockwise orientation.

Details for the Cost function

Error function f_1 weights the (potentially large) intersection of two large sets much stronger than the (potentially small) intersection of two small sets or a small with a large set. Since the intersection size of a

sequence of sets is restricted by its smallest set, we propose to normalize the partial errors by this upper bound. Furthermore f_1 does not take into account that a barely visible intersection (where an intersection should be present) is worse than a little too much graphical overlap. An error function compensating for those two effects can be defined by

$$f_2(I) = \frac{d(G(I), |A(I)|)}{\min_{i \in I} |A_i|} \begin{cases} \alpha & \text{if } A(I) = \emptyset \\ \beta & \text{if } A(I) \neq \emptyset,\ G(I) < |A(I)| \\ \gamma & \text{otherwise} \end{cases} . \tag{1}$$

A schematic view of eqn. 1 is shown in Figure S2.

Figure S2 Error function: weighting of the partial errors

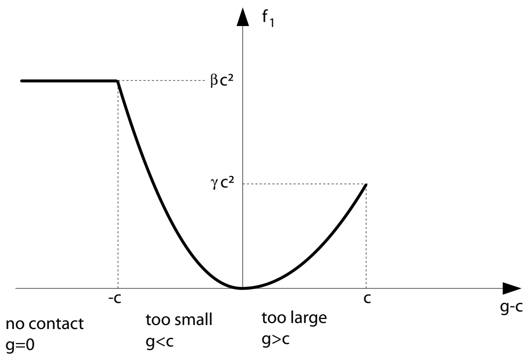

Figure S2: Error function f_2 (eqn. 1) for $c > 0$ and $d(g, c) = (g - c)^2$ showing the three different cases of "no contact" ($g = 0$), "distance too small" ($g < c$), and "distance too large" ($g > c$).

Some details on particle swarm optimization

A swarm consists of a number N of interacting particles such that each particle $j = 1 \dots N$ represents a solution $\mathbf{x}^{(j)} \in \mathbb{R}^n$ in the n-dimensional space having fitness $f(\mathbf{x}^{(j)})$. Additionally, each particle has a velocity vector $\mathbf{v}^{(j)} \in \mathbb{R}^n$ that specifies its current movement in space for each axis. See Figure S3 for a visualization of the update process.

Figure S3 - Particle swarm optimization

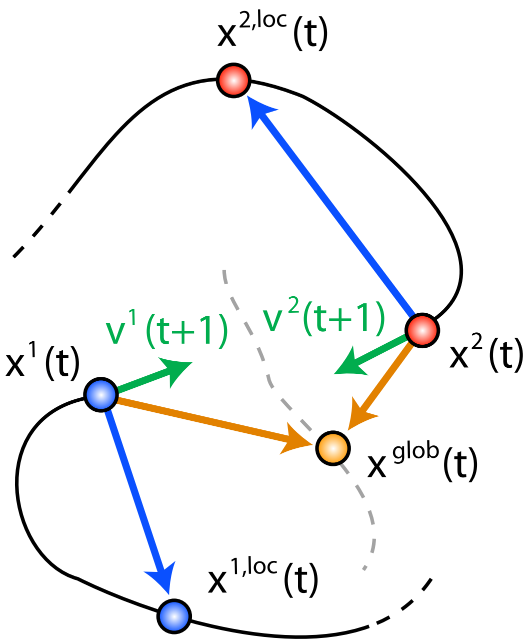

Figure S3: Two particles (circles, $\mathbf{x}^1$ and $\mathbf{x}^2$) surround the global optimum $\mathbf{x}^{glob}$ (orange filled disk). A particle denotes an Euler configuration. The orientation of each particle at time $t+1$ is determined by the current best global and local ($\mathbf{x}^{1,loc}$ and $\mathbf{x}^{2,loc}$) solution.

Software implementation details

For polygons, a single cost function evaluation requires $O(Ln2^{n-1})$ steps for all set combinations. In the restricted case (only intersections of $\leq K < n$ groups are observed) this reduces to $O(L\sum_{k=2}^{K} k\binom{n}{k})$ steps, with L being the number of polygon edges. This can be seen using a tree structured decomposition (Figure S4) of all the $2^n - 1$ possible combinations of set intersections which ensures that no intersection will be computed twice. The proposed structure was directly implemented into the software as data structure for representing set intersections in combination to the graphical representation.

We define a binary tree with n levels, each representing one set $A_1 \dots A_n$ and its corresponding graphical representation (polygon or circle), so that every right branch at level i implies that set A_i is included into the intersection ($i \in I$) and every left branch implies that the set is not included ($i \notin I$). Only the nodes having a parent leading from a right branch account for the computational complexity - the other nodes are just pointers to their parent nodes. Let $\pi(v) = \langle r_1 \dots r_\ell \rangle$ be a path from the root node v_0 to a node

$v \in V$ in level ℓ. $r_i \in \{L, R\}$ represents the decision whether to follow the left ($r_i = L$) or the right branch ($r_i = R$) at level $i \leq \ell$. Now the corresponding set (the graphical representation $G(v)$ is analogously defined) associated with the node v is $A(v) = \left(\bigcap_{1\leq i\leq \ell,\ r_i=R} A_i\right)$. The number of involved sets at node v is the number of right turns in the tree. At each active node (those with a right branch parent) the true set intersection and its corresponding graphical intersection can be computed using the values of the parent node and the set representing the current tree level. So each intersection is reused and never calculated twice. The sum of all partial errors of all active nodes is exactly E. The tree may be pruned to have at most K right turns for the reduced variant (see text methods section).

Figure S4 - Implementation: Data structure

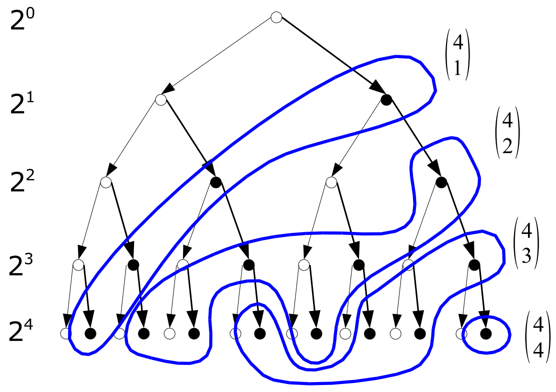

Figure S4: The diagram shows the data structure containing the intersection sets and the corresponding graphical objects. Each level $i = 1 \ldots 4$ contains only the combinations involving the set A_i. The active nodes are marked with black circles. The unfilled circles represent only empty placeholder nodes with pointers to their parents. Blue curves mark the active nodes involving $k = 1, 2, 3, 4$ sets (k right turns). If a grahpical structure involving the intersection of $k-1$ sets with a further set requires $g(k)$ steps then the total number of steps required to build the whole tree is $\sum_{k=1}^{n} \binom{n}{k} g(k)$.

References

1. Buchholz M, Kestler HA, Holzmann K, Ellenrieder V, Schneiderhan W, Siech M, Adler G, Bachem MG, Gress TM: **Transcriptome analysis of human hepatic and pancreatic staellate cells: Evidence for common cell lineage and function**. *J Molecular Medicine* 2005, **83**:795–805. [MB and HAK contributed equally].
2. Zeeberg BR, Qin H, Narasimhan S, Sunshine M, Cao H, Kane DW, Reimers M, Stephens RM, Bryant D, Burt SK, Elnekave E, Hari DM, Wynn TA, Cunningham-Rundles C, Stewart DM, Nelson D, Weinstein J: **High-Throughput GoMiner, an 'industrial-strength' integrative gene ontology tool for interpretation of multiple-microarray experiments, with application to studies of Common Variable Immune Deficiency (CVID)**. *BMC Bioinformatics* 2005, **6**(168).
3. O'Rourke J: *Computational Geometry in C*. Cambridge University Press, second edition 2000.
4. Harris JW, Stocker H: *Handbook of Mathematics and Computational Science*. New York: Springer Verlag 1998.
